# Determinants of dropout and compliance of children participating in a multidisciplinary intervention programme for overweight and obesity in socially deprived areas

**DOI:** 10.1093/fampra/cmac100

**Published:** 2022-09-19

**Authors:** Hevy Hassan, Selinde Snoeck Henkemans, Jolande van Teeffelen, Kees Kornelisse, Patrick J E Bindels, Bart W Koes, Marienke van Middelkoop

**Affiliations:** Department of General Practice, Erasmus MC University Medical Centre Rotterdam, PO Box 2040, 3000 CA Rotterdam, The Netherlands; Department of General Practice, Erasmus MC University Medical Centre Rotterdam, PO Box 2040, 3000 CA Rotterdam, The Netherlands; Dietician Practice in Primary Care, diëtistenpraktijk HRC, Rotterdam, The Netherlands; Fysiotherapie Heemraadssingel, Rotterdam, The Netherlands; Department of General Practice, Erasmus MC University Medical Centre Rotterdam, PO Box 2040, 3000 CA Rotterdam, The Netherlands; Department of General Practice, Erasmus MC University Medical Centre Rotterdam, PO Box 2040, 3000 CA Rotterdam, The Netherlands; Department of General Practice, Erasmus MC University Medical Centre Rotterdam, PO Box 2040, 3000 CA Rotterdam, The Netherlands

**Keywords:** childhood obesity, nutrition/diet, obesity, physical activity/exercise, primary care

## Abstract

**Background:**

Children with overweight and obesity in socially deprived areas (SDAs) are less likely to complete and be compliant to a weight-loss programme.

**Objectives:**

To identify factors associated with dropout and compliance of a multidisciplinary weight-loss programme in SDA.

**Methods:**

This prospective longitudinal cohort study included children (6–12 years) with overweight and obesity in a 12-week multidisciplinary intervention living in SDA in Rotterdam, the Netherlands. Potential predictive variables for dropout and compliance included were age, sex, the weight of the child and parents, quality of life, and referral status (self-registration or referral). A Cox proportional hazards model was performed to study the association between dropout and its potential predictive variables, whereas logistic regression analyses were used for the potential predictors for compliance.

**Results:**

A total of 121 children started the intervention programme. Forty-one (33.9%) children dropped out and 68 (56.2%) were compliant with the intervention. The risk of dropping out of the intervention was significantly lower for a child with overweight parents than for those with parents with normal weight (adjusted hazard ratio [HR] 0.22 [95% confidence interval, CI 0.063–0.75]), and for those with parents with obesity (adjusted HR 0.18 [95% CI 0.060–0.52]). No other potential predictive variables were associated with dropout or compliance.

**Conclusion:**

Children from SDA participating in a weight-loss programme have a relatively high dropout and a low compliance rate. Parental weight seems to be an important predictor for dropout of children from SDA, where children with normal weight or obese parents have the highest risk of dropout compared with children of overweight parents.

**Key messages**

- A high dropout and a low compliance were seen.
- Parental weight seems to be an important predictor for dropout.
- Children with normal weight/obese parents have the highest risk of dropout.
- Future research is needed regarding the reasons of dropout.

## Introduction

Various multidisciplinary weight-loss intervention programmes have been initiated aiming to decrease the prevalence of paediatric obesity.^1^ However, intervention programmes for obese children have often only resulted in small and short-term changes regarding weight status.^2^ The limited effectiveness of these intervention programmes is often thought to be the result of low compliance^3–6^ and high dropout rates.^7^

Compliance is the overall attendance of a child to an intervention programme, while dropout is defined as children who prematurely disengaged. Compliant children have been found to attain a significantly larger reduction in body mass index (BMI)^4,8^ and waist circumference^6^ than their noncompliant peers.

Dropout and noncompliance both increase healthcare costs due to inefficient use of resources.^9–11^ In addition, dropout discourages families to re-enter a weight-loss programme in the future.^9,10^ Given these interactions and consequences, there is a high need to reduce dropout rates and to optimize compliance in intervention programmes targeting children with overweight and obesity.

Particularly low attendance rates of intervention programmes are reported in children with overweight and obesity in socially deprived areas (SDAs).^5^ While, the prevalence of childhood obesity is relatively high in deprived areas.^12,13,14^ Targeted interventions directed at these lower socioeconomic groups are therefore needed. More insight into factors associated with compliance and dropout may help to develop and improve these interventions. Previous research showed multiple predictors of dropout and compliance in multidisciplinary interventions for children who are overweight or obese. Reported predictors for dropout are older age,^4,16,17^ ethnicity,^17,18^ a higher baseline BMI,^18^ and overweight or obese parents^13^ or siblings.^16^ Furthermore, children whose parents have low motivation^10^ are more at risk of dropping out. Similarly, predictors of noncompliance, such as low family income and ethnicity^12^ have been identified. While multiple predictors of dropout and noncompliance for multidisciplinary interventions regarding weight loss in children who are overweight or obese have already been established, research specifically focussing on SDAs is lacking. Therefore, the aim of this study was to identify factors associated with dropout and compliance in a multidisciplinary weight-loss intervention programme for children with overweight or obesity in SDAs in Rotterdam, The Netherlands.

## Methods

### Study design and participants

For the present study, data from a prospective longitudinal cohort conducted among children who were registered and started the Kids4Fit intervention programme was used. Kids4Fit is a 12-week multidisciplinary intervention programme for overweight and obese children living in SDAs in Rotterdam, The Netherlands.^6^ The programme was carried out at 4 locations in deprived areas, where often children of ethnic minorities and low social economic status live. More details are reported elsewhere.^6^

Children aged 6–12 years who were overweight or obese and registered to the Kids4Fit programme between October 2012 and August 2014 were included in the study. Exclusion criteria for participation were underlying medical pathologies, comorbidities, and inability to function in a group. All parents, and children aged 12 years, provided written informed consent before taking the first measurements.

Children were referred to the intervention by healthcare professionals, e.g. general practitioners, dietitians, paediatricians, youth healthcare workers, or self-subscribed. Children who signed up were placed on a waiting list. As soon as in 1 location a group of 8–12 children could be formed, children on the waiting list were able to start the programme.

Data collection was performed at 4 time-points: when children signed up (baseline), at the start of the programme (T1), at the end of the 12-week intervention (T2), and 52 weeks after the start of Kids4Fit (T3).

**Fig. 1.**
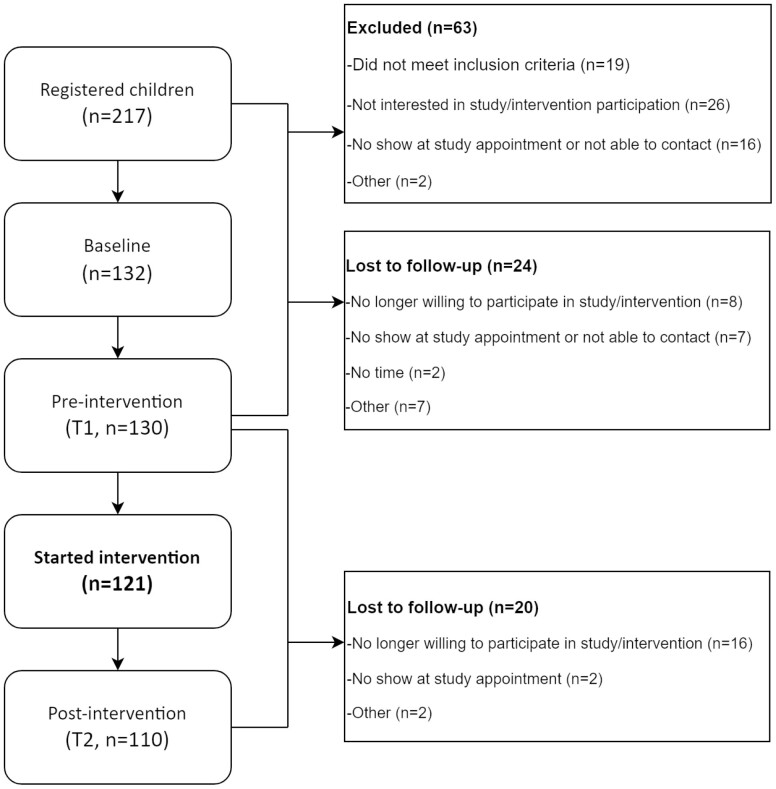
Flow chart of participants who signed up for the Kids4Fit intervention programme (2012–2014).

For the current study purpose, we used preintervention data (measured at T1) for the analyses and therefore only included children who actually started with the intervention (Fig. 1).

### Intervention

Each child and its parents received information on the intervention during an individual intake session with the treatment providers before start of the programme.

The Kids4Fit intervention consisted of child group sessions during a 12-week intervention period: eighteen sessions with a physiotherapist, 4 sessions with a dietician, and 4 sessions with a child psychologist. The sessions with the child psychologist were also assumed to be attended by the parents.

### Measurements

The questionnaire for parents requested information on potential predictors, parental education (high [at least bachelor level] and low [up to secondary school level]), ethnicity (both parents born in the Netherlands, at least 1 parent born abroad), referral (signed up to Kids4Fit on own initiative or referred to Kids4Fit by healthcare provider), previous attempts made to reduce the child’s overweight under supervision (yes, no), parent is willing to change own lifestyle (yes, no), and weight status of the mother (normal weight [BMI ≤24.9], overweight [BMI 25.0–29.9], (morbid) obesity [BMI ≥30.0]). If the weight status of the mother was not available, anthropometric measurements of the father were used instead.

The height and weight of the participating children were measured at all 4 time-points. The height was measured to the nearest 0.1 cm (SECA 217 freestanding mobile stadiometer) and weight to the nearest 0.1 kg (SECA 716 weighing scale). BMI-z scores were calculated with World Health Organization (WHO) reference data from height and weight measures.^19^ Furthermore, information was gathered on physical activity level of the child (inactive [<3 days ≥60 min of exercise] or active [exercise ≥60 min each day]),^20^ eating breakfast (7 days per week).

Self- and proxy-report (in children below the age of 8 years) Health-Related Quality of Life (HRQoL) questionnaires were filled out^20,21^ to evaluate HRQoL in children. The global HRQoL score is given by the sum of all 23 items informing on the physical (8 items), emotional (5 items), social (5 items), and school functioning (5 items) of the child. Higher scoring children (scale range 0–100) have a better HRQoL. For the current study, the total HRQoL was used.

Treatment providers registered attendance during the 12-week intervention. The start of the intervention is the first consult with the physiotherapist, i.e. the first attendance date. Based on the first attendance date, “time-to-event” was calculated, measured from the start of the intervention to the event, i.e. dropout. Time-to-event was transformed into weeks. Children who prematurely disengaged from the intervention programme were considered dropouts, i.e. children who stopped before the end of the 12-week intervention programme. For the secondary outcome, children with a ≥75% overall attendance rate were regarded compliant.

### Statistics

Data analysis was performed using SPSS (version 25.0). Statistical significance was set at *P* < .05. Descriptive statistics were used to present preintervention characteristics of children who started the intervention. Cox regression was performed for the outcome dropout and logistic regression for the outcome compliance. For both models, univariate and multivariate analyses were performed using the variables age, sex, BMI-z, HRQoL, parental weight status, and subscription to the intervention (signed up on own initiative or referred).

Missing values at T1 were imputed with baseline data for HRQoL scores (8 values imputed), attending sports club (6 values imputed), eating breakfast (7 values imputed), and whether parents were willing to change their lifestyle (8 values imputed). For parental BMI missing data were also imputed using data from baseline, and if not available data from T2 or T3 was used (17 values imputed).

To generate survival curves for graphic representation, BMI-z scores were categorized into overweight and obesity using the WHO Growth Reference for school-age children.^22^ Children with a BMI-z score >1 were classified as overweight and a BMI-z score >2 corresponded with obesity.

## Results

Two hundred and seventeen children registered for the intervention between October 2012 and August 2014. Of the registered children, 63 were excluded (did not want to participate or did not met the inclusion criteria) and 44 lost to follow-up during the period they were on the waiting list (Fig. 1). A total of 121 children started the intervention and were therefore included in the present study (Fig. 1). Characteristics of the study population are shown in Table 1. The mean (SD) age of children was 8.9 (1.8 years) and 40.5% were boys. In most families (79.3%) at least 1 parent was born outside the Netherlands.

**Table 1.** Characteristics of the study population (2012–2014).

	Total (*n* = 121)	Dropout of intervention (*n* = 41)	Completed intervention (*n* = 80)
Age in years [mean (SD)]	8.9 (1.8)	8.7 (1.8)	9.0 (1.8)
Sex (boy)	49 (40.5)	15 (36.6)	34 (42.5)
BMI-z child [mean (SD)]	2.7 (0.7)	2.7 (0.6)	2.7 (0.8)
Overweight or obese siblings	27 (22.3)	7 (17.5)	20 (26.3)
Unknown	5 (4.1)		
Attends sports club	34 (28.1)	10 (24.4)	24 (30.4)
Physical activity			
Active (exercise ≥60 min each day)	26 (21.5)	9 (22.0)	17 (21.5)
Inactive (<3 days ≥60 min of exercise)	24 (19.8)	5 (12.2)	19 (24.1)
Eating breakfast 7 days per week	89 (73.6)		
Unknown	1 (0.8)	31 (75.6)	58 (73.4)
HRQoL—global score [median (IQR)]	80.4 (17.4)	82.6 (14.7)	79.3 (20.7)
Physical score [median (IQR)]	81.3 (25.0)	84.4 (17.2)	81.3 (28.1)
Psychosocial score [median (IQR)]	80.0 (19.6)	80.0 (19.2)	80.0 (21.7)
Ethnicity			
Both parents born in the Netherlands	14 (11.6)	5 (13.9)	9 (12.2)
At least 1 parent born outside the Netherlands	96 (79.3)	31 (86.1)	65 (87.8)
Parental education			
High (at least bachelor level)	16 (13.2)	4 (10.0)	12 (15.4)
Low (up to secondary level)	102 (84.3)	36 (90.0)	66 (84.6)
BMI parent (mother^a^, or if not available father^b^) [mean (SD)]	30.8 (6.2)		
Signed up on their own or referred			
Parent signed up to Kids4Fit on their own initiative	28 (23.1)	6 (15.4)	22 (27.8)
Referred to Kids4Fit by healthcare provider	90 (74.4)	33 (84.6)	57 (72.2)
Previous attempts have been made to reduce the child’s overweight under supervision	68 (56.2)	24 (60.0)	44 (57.9)
Parent is willing to change own lifestyle	110 (90.9)	36 (87.8)	74 (93.7)

*n*(%), unless otherwise stated. Missing values of the total study intervention. Overweight or obese siblings, *N* = 5; attends sports club, *N* = 1; physical activity, *N* = 1; eating breakfast, *N* = 1; ethnicity, *N* = 11; parental education, *N* = 3; signed up on their own or referred, *N* = 3; previous attempts, *N* = 5; parent is willing to change own lifestyle, *N* = 1.

^a^Mother 79%.

^b^Father 21%.

During the 12-week intervention period, 41 (33.9%) children dropped out of the intervention programme, and 68 (56.2%) were compliant with the intervention. Compliance was highest with child psychologist sessions (63.6%) and lowest for physiotherapist sessions (52.1%).

Of the parents, 61 (50.4%) attended ≥75% of child psychologist sessions.

**Fig. 2.**
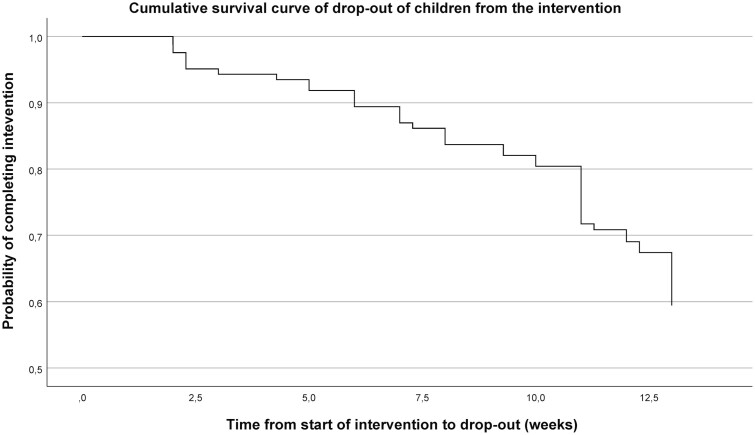
Survival function of dropout of children (dietician, physiotherapist, and child psychologist sessions). Dependent variable, dropout = yes (2012–2014).

Figure 2 shows the overall Cox survival curve for dropout of children during the intervention. The univariate and multivariate Cox proportional hazards models showed an association between parental BMI and the child’s hazard of dropping out (Table 2), in which parents with overweight was the reference group for both parents with normal weight, and obesity. The hazard of dropping out of the intervention was significantly lower in a child with overweight parents than for those with parents with normal weight (adjusted hazard ratio [HR] 0.22 [95% confidence interval, CI 0.063–0.75]) and for those with parents with (morbid) obesity (adjusted HR 0.18 [95% CI 0.060–0.52]). In addition, none of the other factors were associated with dropout of the child.

**Table 2.** Cox regression for dropout of children from the multidisciplinary (2012–2014).

	Univariate analysis HR (95% CI)	Multivariate analysis HR (95% CI)
Age	0.94 (0.79–1.12)	0.91 (0.72–1.15)
Sex (boy)	0.82 (0.44–1.55)	0.75 (0.34–1.68)
BMI-z	1.03 (0.67–1.57)	0.82 (0.43–1.55)
HRQoL total score	1.02 (0.99–1.05)	1.02 (0.99–1.05)
BMI parent categorized (ref. group overweight)		
Normal weight^*^	0.27 (0.086–0.85)^**^	0.22 (0.063–0.75)^**^
(Morbid) obese^*^	0.22 (0.085–0.59)^**^	0.18 (0.060–0.52) ^**^
Signed up on their own or referred (signed up on own)	0.54 (0.23–1.30)	0.46 (0.17–1.24)

^*^Reference group is overweight.

^**^Significant *P* < 0.5.

No significant association was found between compliance and any of the possible predictive factors (Table 3).

**Table 3.** Logistic regression for compliance of children to intervention (dietician, physiotherapist, and child psychologist sessions) (dependent variable, compliance = yes), univariate and multivariate analyses (2012–2014).

	Univariate analysis OR (95% CI)	Multivariate analysis OR (95% CI)
Age	1.03 (0.84–1.26)	0.94 (0.72–1.23)
Sex (boy)	1.08 (0.52–2.24)	1.56 (0.60–4.08)
BMI-z	0.76 (0.45–1.29)	0.48 (0.21–1.09)
HRQoL total score	1.01 (0.98–1.04)	1.02 (0.98–1.05)
BMI parent categorized		
Normal weight	2.06 (0.61–6.96)	2.21 (0.62–7.92)
(Morbid) obese	1.92 (0.59–6.21)	2.07 (0.60–7.11)
Signed up on their own or referred (signed up on own)	0.66 (0.28–1.60)	0.48 (0.17–1.36)

OR, odds ratio.

**Fig. 3.**
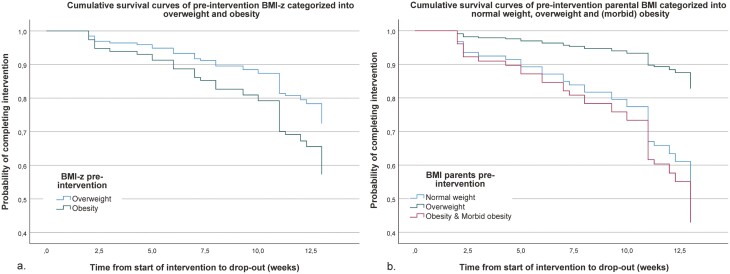
Cox survival curves of dropout of children from the intervention, with BMI-z scores categorized into overweight and obese (a), parental BMI categorized into normal weight, overweight and (morbid) obese (b) (2012–2014).

In all analyses, the BMI-z of the child was not significantly associated with dropout nor with compliance. A graphical representation of the cumulative dropout for BMI-z of the child and parental BMI are shown in Fig. 3.

## Discussion

This study aimed to identify variables associated with dropout and compliance of a multidisciplinary weight-loss intervention programme in SDAs. In our analyses, the only significant association found was between parental BMI and the hazard of dropping out of the child.

In our study, 33.9% of children dropped out, which is slightly lower than the median of 37% found in a review on paediatric obesity management by Dhaliwal et al.^23^ This is noteworthy because children in deprived areas, as compared with nondeprived areas, are usually less likely to complete a weight-loss intervention programme.^5,7,11^ However, there is still substantial room for improvement. Previous research showed that children who have parents who are more involved, adhere better to the intervention and lose significantly more weight.^24^ In our study, parents were expected to attend psychologist sessions, and the compliance for this part of the intervention was low. Therefore, we believe that professionals should try to improve parental involvement in all parts of the interventions, e.g. motivate the parents to participate when they are expected, and to involve the parents more in other parts of the intervention.

Previous literature shows that a higher parental BMI leads to a higher dropout of the child during a weight-loss intervention.^12^ In the present study, parental BMI was categorized (normal weight, overweight, obesity), as there was a nonlinear correlation between the parental weight status and the dropout of the child. A statistically significant lower hazard of dropout for children with overweight parents was seen compared with children with obese parents and parents with normal weight, suggesting that children whose parents are overweight have the lowest risk of dropout from the intervention. We can only speculate about the possible explanation of our findings. One could speculate that normal weight parents may not see the urgency of losing weight and may worry less about weight. Though it is also possible that parents with overweight may not recognize the urgency to lose weight. The higher risk of dropout found in the group of children with obese parents may be explained by the notion that these parents may see the urgency, however, the necessary lifestyle adjustments might be more difficult for them. Since parental weight appears to play a role in the dropout of the child during an intervention programme, a customized programme that fits the parents’ weight category may reduce the dropout of the child. A possible adjustment might be to offer information adapted to the weight status of the parents, and as mentioned before to motivate parents to (actively) participate during the child’s intervention. However, further qualitative research should therefore aim to get insight into reasons of dropout within families with different weight status.

Pott et al. showed that children who have parents with low motivation were more at risk of dropping out.^16^ Surprisingly, we found no association between the motivation of parents and dropping out of the child in our study. This may be due to the threshold for motivation as families had to show high motivation at the start of the Kids4Fit programme, parents who signed up on their own initiative might have been even more intrinsically motivated than their referred peers, since they must have been actively looking for ways to reduce the weight of their child. Absence of significance might be also due to the relatively small sample size, resulting in a large CI.

No associations were found for the outcome compliance. However, previous research showed that children with higher baseline BMI were less likely to be compliant than those with lower BMI.^24^ This is a reason for concern, since participating and completing a weight-loss intervention is even more crucial for children with a higher BMI.

Previous qualitative studies^18^ have pointed out that the complexity of daily life, e.g. lack of financial resources and busy daily schedule, is an important barrier for compliance and dropout of interventions.^24,25^ In this study, these factors were unfortunately not available and therefore could not be taken into consideration, and it therefore remains unknown if these factors play a role in this specific study population.

### Strength and limitations

A strength of our study is that we evaluated determinants of compliance and dropout of an existing weight-loss intervention that runs specifically in SDAs. Research in SDAs is still limited and often challenging because people who have a low socioeconomic status can be more difficult to reach.^26^

Due to the relatively small sample size, our study had limited power to detect differences between the variables and our outcomes (e.g. dropout and compliance), even though associations might have been present. For example, only 28 parents signed up on their own initiative compared with 90 families who were referred by a healthcare professional. Due to the small sample size, the rule of 1 in 10 could not be applied,^27^ since the chosen variables were considered to be too important to exclude.

In addition, information on weight and height of both mothers and fathers was asked, but most times only that of the mother was reported back. Therefore, we used mother’s BMI in most of the cases, and only if not available that of the father.

## Conclusion

Children from SDAs participating in a weight-loss programme have a relatively high dropout and noncompliance rate. Parental BMI seems to be an important predictor for dropout of children from deprived areas, where children with normal weight or obese parents have the highest risk of dropout compared with children of overweight parents. To improve compliance and reduce dropout of the children, the importance of the intervention should be discussed with the parents at baseline, and during the intervention parents should be more involved in all parts of the programme. If necessary, the intervention may be adjusted to the parental weight status. However, future (qualitative) research is needed regarding the reasons of dropout within families with different weight status.

## Supplementary Material

cmac100_suppl_Supplementary_Checklist

## Data Availability

The data are not publicly available due to privacy or ethical restrictions.
